# Agreements and disagreements regarding “shaken baby syndrome”

**DOI:** 10.1007/s00381-022-05621-5

**Published:** 2022-08-09

**Authors:** Niels Lynøe, Anders Eriksson

**Affiliations:** 1 Centre for Healthcare Ethics, Karolinska Institutet, Stockholm, Sweden; 2 Department of Community Medicine and Rehabilitation, Forensic Medicine, Umeå University, Umeå, Sweden

Dear Editor:

We agree with some of the statements by Vinchon et al. [1], e.g., that (i) circular reasoning is associated with a high risk of bias in diagnostic accuracy studies and that (ii) confession studies might represent a possibility to avoid a high risk of bias through circular reasoning—if the confessions are documented, reliable, detailed, and not associated with pressure from the judicial system.

We also agree that (iii) a legal acquittal does not prove a wrong diagnosis, but of course it is just as true that a conviction does not prove a correct diagnosis—especially not if an expert witness promoting the traditional shaken baby hypothesis has been involved in the legal procedure.

Furthermore, we surely agree (iv) with the self-evident view that an adult should never shake an infant and have of course never indicated that shaking an infant is harmless. Preventive “do not shake a baby” programs lack, however, significant effects upon the incidence of AHT cases, at least after 2009 [2], and this is why we suggested that scientists should abstain from conflating the three main reasons for shaking: due to crying (51%), due to an apparently life-threatening event (ALTE) (37%), and due to anger or frustration (12%) [3]. Two of these three stated reasons might be preventable, but a caregiver’s spontaneous reaction in an ALTE-like situation, i.e., shaking the infant after its collapse as a revival attempt, is hardly preventable (or even desired to be prevented). Conflating non-preventable and possibly preventable shaking might dilute a possible preventive effect upon the latter group, and we appeal that Vinchon et al. separate shaking due to a revival attempt from other reasons for shaking, in accordance with other authors [4, 5]. Astonishingly, however, Vinchon et al. claim that they have “no recollection of malaise followed by shaking as a reviving method”! Nevertheless, cases subjected to a revival attempt due to ALTE are usually classified as AHT [3–5].

Still a few important issues must, however, be discussed further in relation to the statements by Vinchon et al. [1] and the deep scientific controversy regarding “shaken baby syndrome.”

## False confessions

To take the point of departure in an assumption, a caregiver always lies if he/she does not give “an ‘acceptable’ explanation” does not qualify as a scientific approach. The authors’ general assumption that “perpetrators confess in order to relieve their conscience, based on the fact that there is no benefit for them to do so” completely ignores the vast literature on false confessions. Furthermore, a caring and deeply worried parent will desperately try to find an explanation to an unexplainable illness of his/her child and might thereby exaggerate insignificant events—which by some has been interpreted as a “confession.” Again, this stresses the need to carefully document the circumstances under which a “confession” is given, as well as the exact content of this “confession.”

## The terms “shaken baby syndrome” (SBS) and “abusive head trauma” (AHT)

Traumatic shaking of infants undoubtedly occurs, but the term “SBS” is a misnomer for several reasons, among others as it by definition connects a mechanism (shaking) with three non-specific medical findings, the “triad” of subdural hemorrhage, retinal hemorrhages, and encephalopathy—a presumed *causal* relationship which lacks solid scientific support [3, 6, 7]. The American Academy of Pediatrics conflated in 2009 SBS cases into the disparate group of AHT cases [8]. This doubtful step has caused further problems, as these two groups may have different etiologies, pathophysiologies, and mechanisms.

## The terms “insufficient” and “limited” scientific evidence

Using widely accepted scientific methods, standards, and terminology [9], the main conclusion of the SBU report was that there is “insufficient” evidence for predicting that a baby had been shaken based on triad findings only [6]. Hence, Vinchon et al. completely ignore scientific standards when they “(base) the diagnosis of AHI …on medical findings alone.” [1].

Furthermore, in opposition to the well-founded second conclusion of the SBU report that “there is ‘limited’ scientific evidence that the triad and therefore its components can be associated with traumatic shaking,” Vinchon et al. flippantly “strongly disagree with this conclusion” by referring to a handful of cherry-picked—but not quality assessed—references, and to deceptive and undefined “clinical experience”—where the clinician never has access to the “true explanation” of the medical findings. Obviously, the authors [1] are not familiar with the GRADE terminology [9] and, in practice, now seem to ignore the detrimental effects of circular reasoning in previous SBS research [6].

Revealing the serious flaws of the never proven traditional SBS theory [6] can of course never “undermine prevention measures for this severe, avoidable and thus unacceptable condition….” [1] On the contrary, scientifically valid conclusions must be based on evidence and never upon such obviously value-based goals. Pediatric practitioners must therefore learn to accept the different requirements upon clinical work versus scientific work and upon clinical work versus the task of a medico-legal expert. Vinchon’s earlier statement “We admit that we were a bit disturbed to find a 100% positive predictive value for the association of severe RH with subdural hematoma (SDH) and absence of signs of impact, because this figure does not look like a scientific result; however, from a legal perspective, we think that this is precisely what a judge hopes for” [10] represents an unacceptable approach to the rule of law.

Also, other comments by Vinchon et al. (1) are astonishing and remarkable, e.g., that their co-authoring intensivist has “great experience” of SIDS—whereas in most jurisdictions worldwide such deaths are investigated by (forensic) pathologists! Lastly, it seems necessary to clarify to Vinchon et al. that evidence-based medicine and randomized controlled trials are not synonymous concepts.
